# Understanding the charge transport properties of redox active metal–organic conjugated wires†
†Electronic supplementary information (ESI) available. See DOI: 10.1039/c7sc04727d


**DOI:** 10.1039/c7sc04727d

**Published:** 2018-02-19

**Authors:** Donglei Bu, Yingqi Xiong, Ying Ning Tan, Miao Meng, Paul J. Low, Dai-Bin Kuang, Chun Y. Liu

**Affiliations:** a Department of Chemistry , Jinan University , 601 Huang-Pu Avenue West , Guangzhou 510632 , China . Email: tcyliu@jnu.edu.cn; b School of Molecular Sciences , University of Western Australia , 35 Stirling Highway , Crawley , 6009 , WA , Australia; c School of Chemistry , SunYat-sen University , Guangzhou 510275 , P. R. China

## Abstract

Layer-by-layer assembly of the dirhodium complex [Rh_2_(O_2_CCH_3_)_4_] (Rh_2_) with linear *N*,*N*′-bidentate ligands pyrazine (L_S_) or 1,2-bis(4-pyridyl)ethene (L_L_) on a gold substrate has developed two series of redox active molecular wires, (Rh_2_L_S_)_*n*_@Au and (Rh_2_L_L_)_*n*_@Au (*n* = 1–6). By controlling the number of assembling cycles, the molecular wires in the two series vary systematically in length, as characterized by UV-vis spectroscopy, cyclic voltammetry and atomic force microscopy. The current–voltage characteristics recorded by conductive probe atomic force microscopy indicate a mechanistic transition for charge transport from voltage-driven to electrical field-driven in wires with *n* = 4, irrespective of the nature and length of the wires. Whilst weak length dependence of electrical resistance is observed for both series, (Rh_2_L_L_)_*n*_@Au wires exhibit smaller distance attenuation factors (*β*) in both the tunneling (*β* = 0.044 Å^–1^) and hopping (*β* = 0.003 Å^–1^) regimes, although in (Rh_2_L_S_)_*n*_@Au the electronic coupling between the adjacent Rh_2_ centers is stronger. DFT calculations reveal that these wires have a π-conjugated molecular backbone established through π(Rh_2_)–π(L) orbital interactions, and (Rh_2_L_L_)_*n*_@Au has a smaller energy gap between the filled π*(Rh_2_) and the empty π*(L) orbitals. Thus, for (Rh_2_L_L_)_*n*_@Au, electron hopping across the bridge is facilitated by the decreased metal to ligand charge transfer gap, while in (Rh_2_L_S_)_*n*_@Au the hopping pathway is disfavored likely due to the increased Coulomb repulsion. On this basis, we propose that the super-exchange tunneling and the underlying incoherent hopping are the dominant charge transport mechanisms for shorter (*n* ≤ 4) and longer (*n* > 4) wires, respectively, and the Rh_2_L subunits in mixed-valence states alternately arranged along the wire serve as the hopping sites.

## Abstract 2

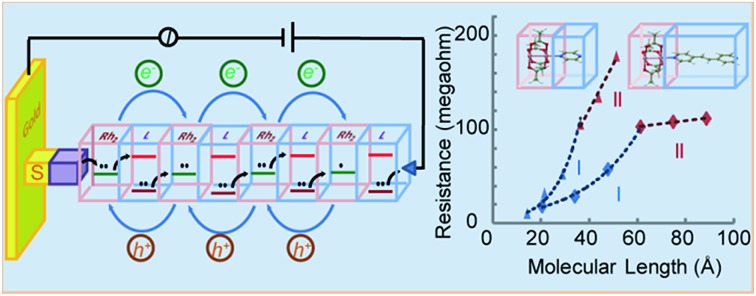
For Rh_2_-organic molecular wires, we found that weaker coupling systems built using longer bridging ligands exhibit better electrical conductance.

## Introduction

Molecular electronics has attracted great attention due to the potential applications of preprogrammed molecules as components in nanoscale circuits.^1^,^2^ Whilst molecular junctions that offer electronic function equivalent to traditional components such as rectifiers,^3^ switches^1^,^4^–^6^ and transistors^7^ receive growing attention, the synthesis of linear molecules that can be embedded between two electrodes and function as molecular wires continues to be of primary importance for the development of science underpinning the operation of molecular electronic devices.^8^–^11^ Generally speaking, the conductive performance of a molecular wire in a molecular junction is determined by a convolution of factors including the environment,^12^–^14^ the nature of the molecule–electrode contacts^15^,^16^ and the electronic configuration of the molecule.^17^,^18^ Detailed knowledge concerning these various structure–functionality relationships within molecular junctions is therefore much in demand before any practical usage of such devices can be realized.^1^,^3^,^8^,^12^,^17^,^19^–^21^ Aiming at this goal, significant efforts have been made to create molecular junctions with various backbones and controlled lengths, which are tested using an increasingly diverse array of electrode–molecule–electrode junction techniques.^19^,^22^–^24^ Organic compounds with π-conjugated backbones have served as models through which to explore charge transport mechanisms and the influence of (un)saturation in the molecular backbone on electrical properties.^17^,^20^,^25^–^27^ Increasingly, chemical functionality is being introduced into prototypical wire-like structures. Redox active molecular wires, linear molecules with one or more periodically inserted redox centers, show weak length-dependence of redox conduction,^27^–^30^ which can be reversibly tuned with a gate electrode.^19^ These features lead to growing interest in the study of molecular wires with integrated metal centers.^31^–^33^

In both single molecular junctions^19^,^34^ and self-assembled monolayers (SAMs),^17^,^20^,^25^–^27^ two principal conductance mechanisms, charge tunneling for shorter wires and charge hopping for longer wires, have been identified. With increasing wire lengths, a switch in the predominant mechanism from tunneling to hopping is observed for wires of 4–6 nm.^17^,^20^,^34^,^35^ These two mechanisms are in accordance with the fundamental studies of electron transfer in donor(D)–bridge(B)–acceptor(A) compounds in solution. It is found that D–A electron transfer across a short bridge proceeds by the super-exchange mechanism, whereas long-distance electron transfer is achieved by sequential hopping.^36^ It is worthwhile to note that in the latter case, the individual hopping steps are interpreted through an underlying super-exchange formalism as well.^36^ Moreover, as is well known, D–A electron transfer kinetics is governed by donor–acceptor electronic coupling, which is tuned by the bridge structure as well as the donor–bridge energy gap.^37^,^38^ Therefore, the through-bond electron transfer in D–B–A systems is intrinsically a resonant behavior of the transferring electron, which necessitates a compatibility of the molecular orbitals in energy and symmetry.^36^ From the similarities in the electron transfer mechanism between D–B–A systems and electrode–molecule–electrode junctions, it would be interesting to validate the understandings on electron transfer in solution and at molecular junctions. Furthermore, to fully understand the hopping mechanism, the chemical nature of the hopping sites for a given molecular system need to be clarified.^19^,^27^ For example, for oligophenyleneimine (OPI) wires, it has been proposed that the charge-hopping site consists of three repeating conjugated subunits.^17^ Unfortunately, for most of the wire systems studied, the hopping sites are not clearly defined, which prevents a deeper understanding of the microscopic process of charge transport.

Investigation of the electron transport characteristics in the two mechanistic regimes and the transition from one to another relies on the development of a series of structurally related wires with precisely controlled lengths, which can be realized conveniently *via* layer-by-layer methods with SAM templates.^17^,^20^,^25^,^26^,^39^ Coordination chemistry has emerged as a particularly useful tool in this regard.^28^,^39^–^41^ In integrating metal complex units into a π-conjugated backbone, covalently bonded dimetal units (M_2_) can be desirable candidates.^40^ With appropriately designed and synthesized M_2_ building blocks and bridging ligands, both axial and equatorial linkages can be realized to construct metal–organic hybrid wires.^8^,^40^ From an equatorial linkage, metal–ligand orbital interactions would generate a d(δ)–p(π) conjugated wire,^42^,^43^ while an axial binding mode gives a wire with π(M_2_)–π(bridge) conjugation.^8^,^44^,^45^ Such well-defined electronic structures should be highly beneficial to the rational design of wire-like compounds and mechanistic study of charge transport.

In the present work, two series of metal–organic hybrid wire-like structures with similar backbones were prepared by the fabrication of the SAMs on gold surfaces through axial coordination of the dirhodium building block [Rh_2_(O_2_CCH_3_)_4_] (Rh_2_) with the conjugated *N*,*N*′-bidentate bridging ligands (L), pyrazine (L_S_) and 1,2-bis(4-pyridyl)ethene (L_L_), and denoted as (Rh_2_L_S_)_*n*_@Au and (Rh_2_L_L_)_*n*_@Au, respectively. By taking a stepwise fabrication approach, the number of Rh_2_L repeating units (*n* = 1–6) was precisely controlled, giving the wire-like structures lengths up to ∼9 nm. These two wire series have similar molecular backbones, but different lengths for the wires with the same number of building blocks, which permit detailed studies of the impact of Rh_2_L building blocks on charge transport properties in the tunneling and hopping regimes. The electron transport characteristics of the resulting SAMs were measured using conductive probe atomic force microscopy (CP-AFM). For both systems, a change of length dependence from an exponential to a linear relationship for the current–voltage (*I*–*V*) characteristics was observed at *n* = 4, which signals a transition in the charge transport mechanism. Notably, smaller *β* values were found for the system having the longer bridging ligand for both short (*n* = 1–4) (*β*_T_ = 0.044 ± 0.002 Å^–1^, verses 0.101 ± 0.012 Å^–1^ for (Rh_2_L_S_)_*n*_@Au) and long (*n* = 4–6) (*β*_H_ = 0.003 ± 0.001 Å^–1^ verses 0.035 ± 0.003 Å^–1^ for (Rh_2_L_S_)_*n*_@Au) junctions, although the electronic coupling within the L_S_ bridged series is significantly stronger. DFT calculations on the Rh_2_L fragments coupled with estimates from spectral data reveal that the 1,2-bis(4-pyridyl)ethene-based (Rh_2_L_L_)_*n*_ wires offer smaller HOMO (Rh_2_ dπ)–LUMO (bridge π*) gaps than the shorter pyrazine-based (Rh_2_L_S_)_*n*_ systems, thus, accounting for the improved charge hopping of this system. These experimental and theoretical results suggest that in these (Rh_2_L)_*n*_ wire systems the Rh_2_L complex units serve as the hopping sites for charge hopping over a long distance.

## Results and discussion

### Fabrication of the (Rh_2_L)_*n*_@Au

**Fig. 1:**
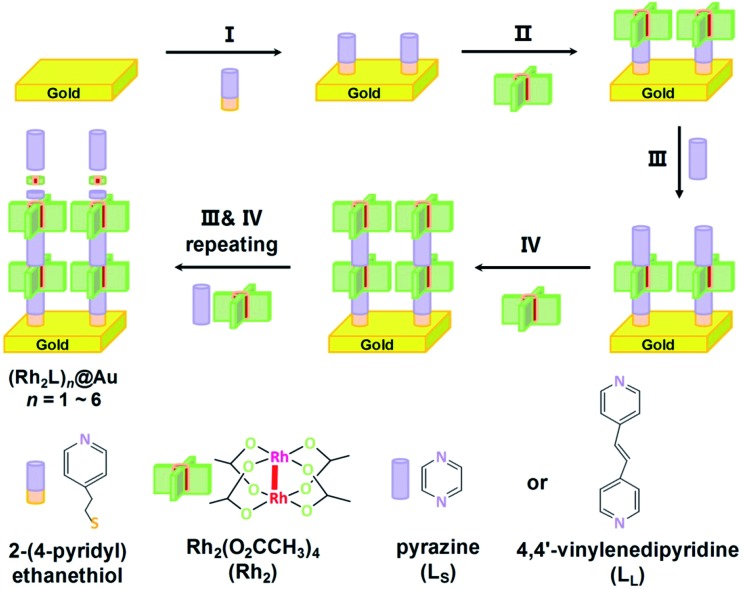
Schematic description of the stepwise assembly of the metal–organic hybrid SAMs on Au substrates, (Rh_2_Ls)_*n*_@Au and (Rh_2_L_L_)_*n*_@Au. Step I, assembling the pyridyl-terminated anchor (2-(4-pyridyl) ethanethiol) on the gold surface. Step II, implanting the first Rh_2_ building block. Step III, introducing a bridging ligand. Step IV, adding an Rh_2_ building block.

The fabrication process of the Rh_2_-ligand SAMs is schematically described in Fig. 1. As is well known, the dirhodium complex [Rh_2_(O_2_CCH_3_)_4_] is a strong Lewis acid with respect to its axial coordination capability and readily forms [Rh_2_(μ-O_2_CCH_3_)_4_L_2_] adducts with Lewis bases, L, and is stable under aerobic conditions. These features allow the facile assembly of Rh_2_ and linear *N*,*N*′-bidentate bridging ligands L (L = L_S_ for pyrazine or L_L_ for 1,2-bis(4-pyridyl)ethene), yielding a linear structure with the Rh–Rh bonds aligned with the long molecular axis. To immobilize the SAMs onto the gold substrate, 2-(4-pyridyl) ethanethiol was utilized as a molecular anchor that is bonded to the Au surface with the S atom, preparing a pyridyl group for the complexation of the incoming Rh_2_ building block. Similar processes involving [Rh_2_(phen)_2_(μ-O_2_CCH_3_)_2_(NCMe)_2_], pyrazine (L_S_) and 4-thiopyridine as a surface anchor,^46^ and systems consisting of bis(Rh_2_) dimers, 1,2-bis(4-pyridyl)ethene (L_L_) and 2-(4-pyridyl) ethanethiol as anchors^40^ have been reported by other groups.

Here, the assembly process started with the immersion of the gold substrate in an ethanol solution of 2-(4-pyridyl) ethanethiol (0.01 mM) for one hour (Step I). This pyridyl-functionalized Au substrate was then soaked in an ethanol solution of [Rh_2_(O_2_CCH_3_)_4_] (0.2 mM), in which the Rh_2_ complex exists as EtOH solvated molecules [Rh_2_(O_2_CCH_3_)_4_(EtOH)_2_], at –15 °C for 1 hour. This process allows the first Rh_2_ unit to be immobilized onto the gold surface through metal–ligand complexation (Fig. 1, Step II), forming Rh_2_@Au.^40^ It was found that reactions at room temperature did not afford surface structures of high quality for the subsequent wire growth. In the following step, the functionalized Rh_2_@Au substrate was immersed in an ether solution of the ligand (L = L_S_ or L_L_) for 10 minutes to generate (Rh_2_L)_*n*_@Au (*n* = 1) (Step III). In Step IV a second Rh_2_ moiety was introduced by further reaction with [Rh_2_(O_2_CCH_3_)_4_(EtOH)_2_] (2 minutes). The following steps involve alternate repeating of Steps III and IV with a dipping time of 2 minutes for both processes, which developed the satisfactory metal–organic SAMs on the gold substrates, (Rh_2_L_S_)_*n*_@Au and (Rh_2_L_L_)_*n*_@Au (*n* = 1–6), as indicated by electrochemical and spectroscopic characterization (*vide infra*). After each step, the Au substrates were rinsed with ethanol to remove the excess adsorbates and dried with a stream of N_2_. The fabrication of these monolayers with six Rh_2_L repeating units can be completed in less than three hours, faster than the stepwise fabrication of organic SAMs.^17^,^20^ For (Rh_2_L_S_)_*n*_@Au, with increasing *n* from 2 to 6, the thickness (*T*) of the SAMs increases from 2.04 ± 0.29 to 4.74 ± 0.18 nm, as determined by an AFM based nano-shaving method,^39^ whereas for (Rh_2_L_L_)_*n*_@Au, *T* = 3.08 ± 0.64 nm (*n* = 2) – 8.78 ± 0.53 nm (*n* = 6). These data are in good agreement with the wire lengths (*L*) estimated from the X-ray structures of similar structural motifs (Fig. S4 and S5†).^47^–^49^

### Spectroscopic characterization and properties

**Fig. 2:**
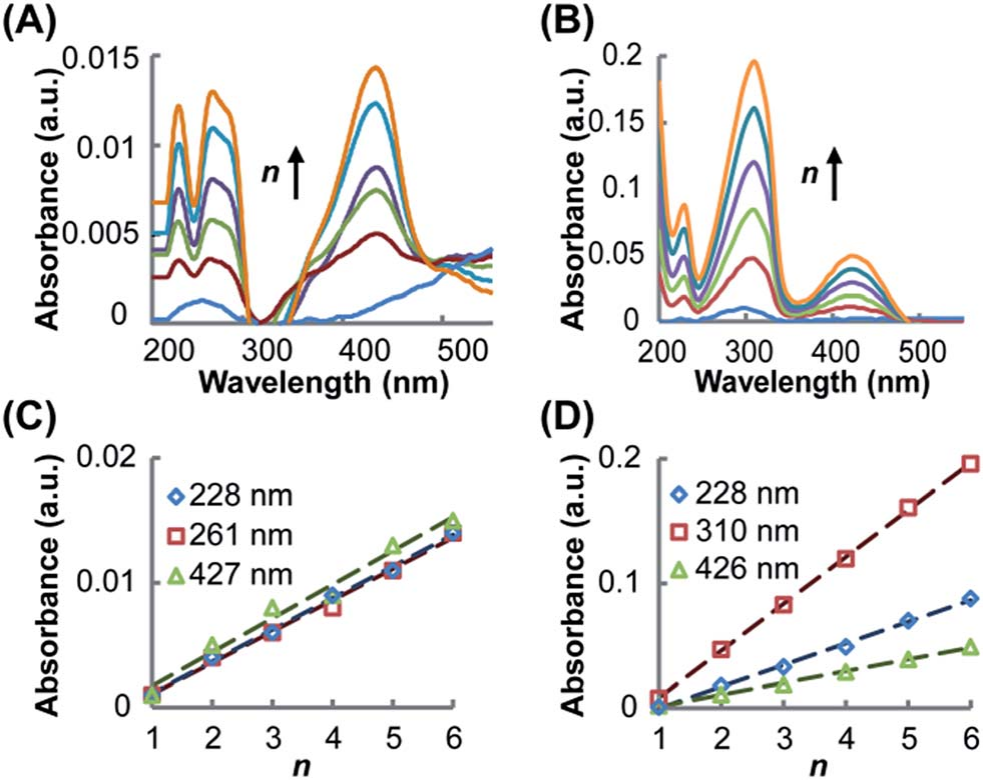
UV-vis spectra of (Rh_2_L_S_)_*n*_@Au (A) and (Rh_2_L_L_)_*n*_@Au (B), showing the increase of band intensity with increasing the number of layers *n* from 1 to 6 (from bottom to top). Linear relationships between the absorbance and *n* for (Rh_2_L_S_)_*n*_@Au (C) and (Rh_2_L_L_)_*n*_@Au (D), determined from the three major absorption bands in the spectra.

UV-vis spectroscopy was utilized to characterize the resultant SAMs and monitor the growth of the SAMs on the Au substrate. As shown in Fig. 2A for (Rh_2_L_S_)_*n*_@Au and Fig. 2B for (Rh_2_L_L_)_*n*_@Au, while the band profiles remain unchanged, the spectral absorption intensities increase linearly as the number of layers (*n*) increases from 1 to 6, confirming the step-by-step fabrication of (Rh_2_L)_*n*_ (*n* = 1–6) on the Au substrates. The SAMs of both systems exhibit four absorption bands in the range of 200–500 nm, similar to the spectra of the oligomers (Rh_2_L_S_)_*n*_ (Fig. S1A†) and (Rh_2_L_L_)_*n*_ (Fig. S1B†), prepared by mixing [Rh_2_(OCCH_3_)_4_] with the bridging ligands. Comparison of the spectra of the molecules immobilized on the Au substrate with the spectra of the associated oligomers and free bridging ligands (Fig. S1C and D†) provides accurate spectral assignments to the electronic transitions. The low energy absorption band in the spectra (Fig. 2), *ca.* 427 nm (Rh_2_L_S_)_*n*_@Au and 426 nm for (Rh_2_L_L_)_*n*_@Au, is assigned to the π*(Rh–O) → σ*(Rh–O) transition within the [Rh_2_(O_2_CCR)_4_] core,^50^,^51^ which is insensitive to the nature of the axial ligands. The higher energy absorbances (*λ*_max_ < 228 nm) are attributed to transitions occurring within the bridging ligands. In the spectra of the free bridging ligands, an intense absorption band is observed at 250 nm for pyrazine (Fig. S1C†) and at 280 nm for 1,2-bis(4-pyridyl)ethene (Fig. S1D†); for the SAMs on the Au substrate these bands are red shifted to 261 and 310 nm, respectively. These bands arise likely from the π → π* transition in the aromatic system of the ligands. It is notable that for (Rh_2_L)_*n*_@Au and oligomers (Rh_2_L)_*n*_ this band is broad and asymmetrical. There is a shoulder band appearing on the low energy side of this dominant absorbance, *ca.* 285 nm for (Rh_2_L_S_)_*n*_@Au and 330 nm for (Rh_2_L_L_)_*n*_@Au, as shown in Fig. 2A and B, which is not present in the spectra of the free ligands (Fig. S1C and D†). Therefore, these absorption bands are ascribable to the metal (Rh_2_) to ligand (bridge) charge transfer (MLCT). Importantly, (Rh_2_L_S_)_*n*_@Au has a MLCT energy significantly higher than that for (Rh_2_L_L_)_*n*_@Au.

### Electrochemical characterization and properties

**Fig. 3:**
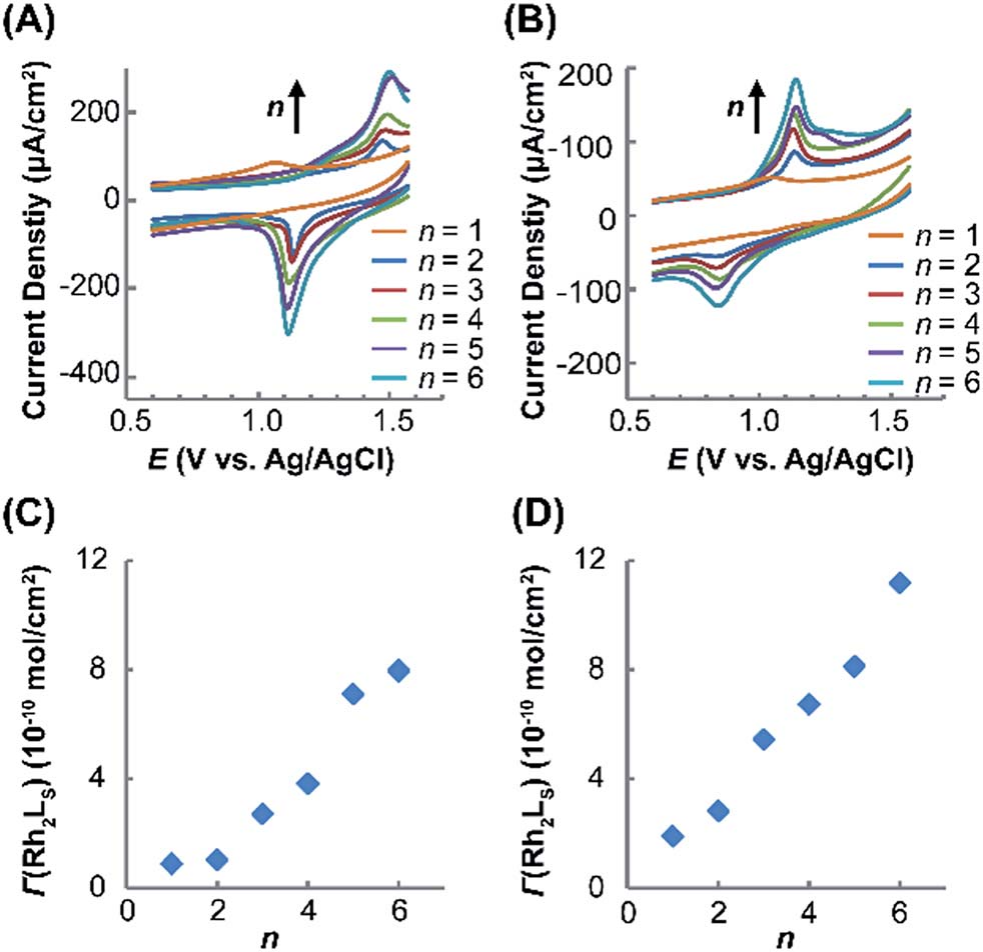
Cyclic voltammograms (CVs) for (Rh_2_L_S_)_*n*_@Au (A) and (Rh_2_L_L_)_*n*_@Au (B) (*n* = 1–6, from inside to outside). (Rh_2_L_S_)_*n*_@Au shows a high half-wave potential and a large anodic–cathodic peak separation, relative to the analogues with longer bridging ligands, which indicates stronger coupling between the neighboring Rh_2_ centers along the wires. Surface coverage varies as a function of *n* for (Rh_2_L_S_)_*n*_@Au (C) and (Rh_2_L_L_)_*n*_@Au (D).

Electrochemical measurements of the immobilized SAMs were performed in CH_2_Cl_2_ with an ^*n*^Bu_4_NPF_6_ electrolyte (1.0 M). The cyclic voltammograms (CVs) obtained for (Rh_2_L_S_)_*n*_@Au and (Rh_2_L_L_)_*n*_@Au with *n* = 1–6 are shown in Fig. 3A and B, respectively. As expected, in the CVs for (Rh_2_L)_*n*_@Au, the current density increases with increasing the number of the Rh_2_ units. By variation of the scan rate from 0.10 to 0.50 V s^–1^ for the CV measurements (Fig. S2†), a linear correlation of the current intensity with the scan rate is observed, indicating that the Rh_2_ units of the linear molecules are surface bounded. The SAMs with a Rh_2_ monolayer (*n* = 1) show a weak redox wave at almost the same potentials, *E*_1/2_ = ∼1.05 V, regardless of the axial coordination of pyrazine or 1,2-bis(4-pyridyl)ethane, and the cathodic and anodic peaks appear at similar potentials (Fig. 3A and B). To validate these potentials for the Rh_2_ redox centers on the SAMs, two reference compounds, Rh_2_(O_2_CCH_3_)_4_(C_5_H_5_N)_2_ and Rh_2_(O_2_CCH_3_)_4_(C_5_H_5_NC_2_H_2_C_6_H_6_)_2_, analogous to the Rh_2_L_2_ complex moieties in (Rh_2_L_S_)_*n*_@Au and (Rh_2_L_L_)_*n*_@Au, respectively, were prepared and the redox properties were examined by cyclic voltammetry in CH_2_Cl_2_ solution. Quasi-reversible CVs (Fig. S3†) were observed for these Rh_2_ complexes, which gave the *E*_1/2_ values of 0.97 V and 0.95 V, respectively, in good agreement with the data for the SAMs of *n* = 1. Interestingly, after additional Rh_2_ layers are introduced onto the SAMs, the cathodic (*E*_pc_) and anodic (*E*_pa_) peaks are largely displaced. Then, the half wave redox potentials (*E*_1/2_), 1.30 V for (Rh_2_L_S_)_*n*_@Au (*n* = 2–6, Fig. 3A) and 1.03 V for (Rh_2_L_L_)_*n*_@Au (*n* = 2–6, Fig. 3B), are estimated from *E*_1/2_ = (*E*_pa_ + *E*_pc_)/2. SAMs (Rh_2_L_L_)_*n*_@Au (*n* = 2–6) show an *E*_1/2_ nearly identical to that of (Rh_2_L_L_)@Au, but a large deviation from 1.05 to 1.30 V is found for the pyrazine bridged series. Therefore, these half-wave potentials (*E*_1/2_) account for the redox process Rh_2_^II/II^ → Rh_2_^II/III^ in the SAMs.

The surface coverage (*Γ*) of the Rh_2_L unit in the SAMs with different Rh_2_L unit numbers (*n*) is determined coulometrically from the redox waves (Table 1), which increases as a function of *n*, as shown in Fig. 3C and D. For (Rh_2_L_L_)_*n*_@Au, the surface coverage of Rh_2_L increases from 1.9 × 10^–10^ mole cm^–2^ to 11.2 × 10^–10^ mole cm^–2^ as *n* increases from 1 to 6. Slightly smaller surface coverage values, *Γ* = 0.9 × 10^–10^ (*n* = 1) – 8.0 × 10^–10^ (*n* = 6) mole cm^–2^, are found for (Rh_2_L_S_)_*n*_@Au. The linear dependence of surface coverage on *n* demonstrates that the monolayers formed on the surface consist of the wires with presubscribed building blocks. These results are fully consistent with the UV-vis spectra, confirming the successful fabrication of the metal–organic wires in the expected manner.

**Table 1:** Selected experimental and calculated data for (Rh_2_L)_*n*_@Au

Monolayer	*E*_1/2_ (V)	*E*_pa_ – *E*_pc_ (V)	*E*_gap_ ^*a*^ (eV)	*Γ* (10^–10^ mol cm^–2^)	*L* (nm)	*T* ^*b*^ (nm)	*R* (10^6^ Ω)	*β*
(Rh_2_L_S_)_1_@Au	1.05	0.02	4.35	0.9	1.43	—	10.3 ± 1.8	0.101 ± 0.012
(Rh_2_L_S_)_2_@Au	1.30	0.38		1.0	2.17	2.04 ± 0.29	31.9 ± 5.5	
(Rh_2_L_S_)_3_@Au				2.7	2.91	—	51.9 ± 4.9	
(Rh_2_L_S_)_4_@Au				3.8	3.65	3.55 ± 0.62	105.7 ± 19.9	0.035 ± 0.003
(Rh_2_L_S_)_5_@Au				7.1	4.39	—	134.0 ± 22.1	
(Rh_2_L_S_)_6_@Au				8.0	5.51	4.74 ± 0.18	178.1 ± 34.5	
(Rh_2_L_L_)_1_@Au	1.05	0.01	3.76	1.9	2.05	—	17.9 ± 9.2	0.044 ± 0.002
(Rh_2_L_L_)_2_@Au	1.03	0.29		2.8	3.41	3.08 ± 0.64	28.9± 5.3	
(Rh_2_L_L_)_3_@Au				5.4	4.77	—	57.4 ± 10.4	
(Rh_2_L_L_)_4_@Au				6.7	6.13	5.83 ± 0.33	102.7 ± 18.9	0.003 ± 0.001
(Rh_2_L_L_)_5_@Au				8.1	7.49	—	107.8 ± 27.3	
(Rh_2_L_L_)_6_@Au				11.2	8.85	8.78 ± 0.53	111.6 ± 29.4	

^*a*^ *E*_gap_ refers to the metal to ligand charge transfer (MLCT) energy.

^*b*^Data collected only for the selected wires to show the length variation with increasing the number of the Rh_2_L units (*n*).

For the Rh_2_ SAMs with *n* = 2–6, the potential hysteresis of reduction is remarkable. These results are in contrast to the observations in most of the redox active SAMs, which have the anodic and cathodic peaks at similar potentials.^39^,^52^ The cathodic–anodic peak separations are also larger than those for SAMs with Rh_2_ complex building blocks (0.14 V) reported in earlier work.^40^ The observations that a shorter bridge gives a larger *E*_pa_ – *E*_pc_ splitting and a larger shift of *E*_1/2_, relative to the potentials for the SAMs with a single Rh_2_ complex unit and the Rh_2_ monomer in solution, indicate that the pronounced redox hysteresis for these two series is somehow related to the electronic coupling between neighboring Rh_2_ centers. Similarly, for moderately coupled mixed-valence (MV) D–B–A systems, we may observe a large *E*_pa_ – *E*_pc_ separation due to the two overlapped potential waves for the two redox centers. In a strong coupling case, organic SAMs constructed from redox active tetrathiafulvalene (TTF) building blocks show two separated redox waves for the two one-electron processes,^20^ resembling strongly coupled MV systems, for example, the Creutz–Taube complex (Δ*E*_1/2_ = 360 mV),^53^ [(NH_3_)_5_Ru (pyrazine)Ru(NH_3_)_5_]^5+^. This correlation of (*E*_pa_ – *E*_pc_) with the electronic coupling between the metal centers is further supported by the Rh_2_ SAMs with a saturated bridging ligand (L = 1,2-dipyridylethane), which exhibit the *E*_pa_ (0.92 V) and *E*_pc_ (0.94 V) peaks at similar potentials.^54^ Therefore, we attribute the larger *E*_pa_ – *E*_pc_ splitting (0.38 V) for (Rh_2_L_S_)_*n*_@Au, in comparison with 0.29 V of (Rh_2_L_L_)_*n*_@Au, to the stronger bridge mediated metal–metal coupling. Similar results were obtained in other metal-containing redox conductive systems. For instance, with phenylene or multi-phenylene bridges, the bis(terpyridyl) metal complex wires showed small *E*_pa_ – *E*_pc_ splitting.^39^ However, the bis(terpyridyl) metal complex networks with a short metal–metal distance exhibit a large potential hysteresis in the electrochemical CV diagrams,^55^ although both systems are constructed with the same metal complex unit.

In molecular junctions, the electronic coupling between two electrodes is a sum of the coupling between the electrode and the wire, and the coupling within the molecule.^56^ Theoretically, the general Hamiltonian of electronic coupling can be expressed as follows:^57^

1 *H* = *H*_mol_ + *H*_electrode_ + *V*

where *H*_mol_ describes the electronic coupling within the molecule, *H*_electrode_ accounts for the electronic coupling between the electrodes and *V* represents the molecule–electrode interaction. When the molecule sandwiched between electrodes is sufficiently large, direct electrode–electrode interaction is weak and the term *H*_electrode_ may be negligible. The term *V* can be evaluated based on the difference in energy between the Fermi level and the highest occupied molecular orbital (HOMO), *i.e.*, *E*_F_ – *E*_HOMO_. Enhanced alignment of the HOMO of metal integrated wires with the gold Fermi level and hence the small *E*_F_ – *E*_HOMO_ gap are well documented.^17^,^20^ From the similar molecular backbones for the two series, similar *E*_F_ – *E*_HOMO_ gaps and thus, similar coupling strength between the electrode and the molecule are expected. Therefore, the electronic coupling of the molecules, as measured from the Rh_2_–Rh_2_ coupling, becomes the key factor that affects the conducting properties of these wires. In the study of D–B–A molecules in solution, it is recognized that there are two major effects contributing to the magnitude of Δ*E*_1/2_ or qualitatively, the coupling strength, that is, electrostatic and resonant effects, resulting from the short D–A distance and strong orbital interactions between D and A, respectively.^58^,^59^ The observed larger reduction hysteresis and greater potential shift in (Rh_2_L_S_)_2–6_@Au indicate the stronger electronic interaction between the redox sites. On the other hand, the unchanged *E*_1/2_ and smaller *E*_pa_ – *E*_pc_ splitting for (Rh_2_L_L_)_2–6_@Au are obviously due to the lengthened bridging ligand that weakens the Rh_2_–Rh_2_ interactions. Therefore, the electrochemical results show that the bridging ligands, the organic parts of the metal–organic hybridized wires, have a substantial impact on the overall coupling (*H*) in the molecular junctions.

### Current (*I*)–voltage (*V*) characteristics

**Fig. 4:**
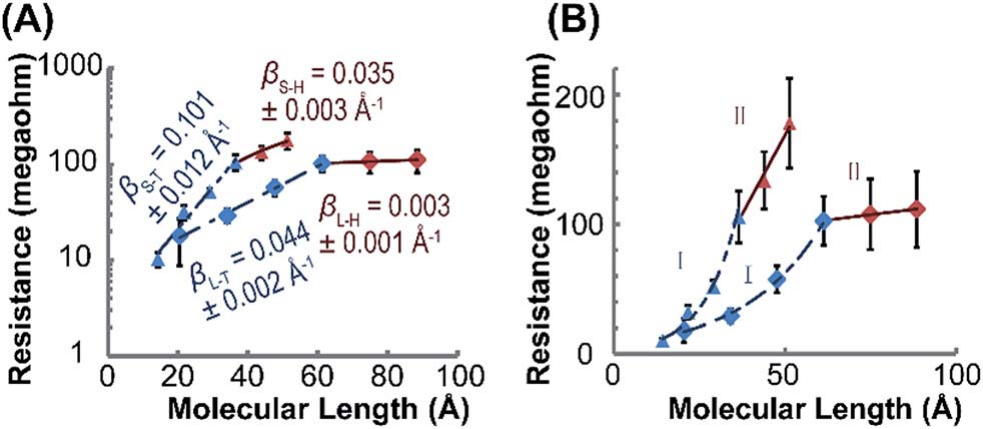
(A) Semi-log plots of resistance *versus* molecular length in regimes I (*n* = 1–4, blue) and II (*n* = 4–6, red) for (Rh_2_L_S_)_*n*_ (triangle) and (Rh_2_L_L_)_*n*_ (diamond). The wire resistances were measured with CP-AFM, in which a Pt/Ir-coated tip was brought into contact with the (Rh_2_L)_*n*_@Au monolayers. The *I*–*V* traces were obtained over ± 0.8 V at a load of ∼1 nN on the tip contact. Each data point is the average resistance obtained from over 30 *I*–*V* traces in the range from –0.4 to +0.4 V. The straight lines are the linear fitting of the data using eqn (2). (B) A linear plot of *R versus L*, demonstrating linear scaling of resistance with the length of the long wires (*n* = 4–6).

The electron transport characteristics of (Rh_2_L_S_)_*n*_@Au and (Rh_2_L_L_)_*n*_@Au were studied using CP-AFM with Pt/Ir AFM probes in contact with the termini of the wires implanted on gold surfaces. The electrical resistances of the molecular junctions formed by Au substrate-wire-AFM probe are determined by taking the reciprocal of *I*–*V* curve slopes and averaging over 30 *I*–*V* traces. Fig. 4 shows a plot of resistance (*R*) *versus* molecular length (*L*) for (Rh_2_L_S_)_*n*_ and (Rh_2_L_L_)_*n*_ (*n* = 1–6). As expected, *R* increases as a function of the wire length. However, here the length-dependence of *R* is best described by a correlation of *R* with the number (*n*) of the Rh_2_L units. As shown in Table 1, similar current resistances (*R*) are found for shorter wires of the two series with *n* ≤ 4. For the longer wires (*n* = 4–6), an abrupt increase of *R* is observed for (Rh_2_L_S_)_*n*_@Au as *n* increases, whereas (Rh_2_L_L_)_*n*_@Au shows a plateau of *R* with adding more Rh_2_L units, even though the wires are largely lengthened, in comparison with (Rh_2_L_S_)_*n*_@Au (Table 1). For both series, an exponential increase of *R* with increasing wire length is observed for systems *n* ≤ 4 (Fig. 4B, regime I), and a linear relationship between *R* and *L* is found for longer structures with *n* = 4–6 (Fig. 4B, regime II). The exponential increase of *R* in regime I is consistent with the characteristic *R*–*L* relationship in the charge tunneling process, described by

2 *R* = *R*_0_ exp (*βL*)

where *R* is the junction resistance, *R*_0_ the effective contact resistance and *β* the distance attenuation factor.^17^,^20^ Exponential fitting to the data for (Rh_2_L_S_)_1–4_ junctions gave an attenuation factor for charge tunneling, *β*_S–T_ = 0.101 ± 0.012 Å^–1^, whereas a smaller attenuation factor, *β*_L–T_ = 0.044 ± 0.002 Å^–1^, is found for the longer wires, (Rh_2_L_L_)_1–4_. Therefore, it is evidenced that these metal–organic wires have relatively small *β* values, compared to organic wires with similar molecular backbones, for example, oligophenylene wires (*β* = 0.61 Å^–1^) and benzylic derivatives of oligophenylene wires (*β* = 0.67 Å^–1^),^60^ oligophenyleneimine (OPI) wires (*β* = 0.3 Å^–1^),^17^ and oligo (ethylene glycol) wires (*β* = 0.24 Å^–1^).^61^ An enhancement of molecular conductance is generally observed by incorporation of metal complex units into the wires.^28^,^30^,^31^,^33^,^62^–^65^ Typical metal–organic wire systems include bis(terpyridine)metal wires^28^,^39^,^52^,^64^,^66^–^68^ with *β* = 0.07–0.001 Å^–1^ and oligo-porphyrin molecular wires with *β* = 0.10–0.03 Å^–1^.^69^–^73^

Different length-dependences of the conductance are observed for the longer wires (*n* = 4–6) in region II, indicating that charge transport occurs *via* a different mechanism. The linear correlation of resistance with length (Fig. 4B) indicates that the hopping mechanism is in operation for charge transport in (Rh_2_L)_4–6_@Au.^19^,^74^ These results indicate a transition in the charge transport mechanism from tunneling to hopping. Tunneling to hopping transition has been observed in several π-conjugated organic systems,^17^,^19^,^20^,^25^,^27^ but scarcely seen in metal–organic wire series.^30^,^31^ It is important to note that for the two series, the transition occurs in wires with the same number of Rh_2_L units (*n* = 4), but with different lengths, *ca.* ∼3.5 nm for (Rh_2_L_S_)_4_@Au and ∼6 nm for (Rh_2_L_L_)_6_@Au. These lengths associated with the change in the conductance mechanism are compatible with other systems.^17^,^19^,^20^,^27^,^28^ For instance, for oligophenyleneimine (OPI) and oligo-tetrathiafulvalene-pyromellitic-diimideimine (OTPI) wires, the turning point from tunneling to hopping is at about 4–5 nm.^17^,^20^ To compare the charge transport characteristics of the wires in different regimes, the *β* parameters in the hopping regime (II) were also derived by exponential fitting of the data (eqn (2)), as reported in the literature.^17^,^22^ *β*_S–H_ = 0.035 ± 0.002 Å^–1^for (Rh_2_L_S_)_4–6_@Au and *β*_L–H_ = 0.003 ± 0.001 Å^–1^ for (Rh_2_L_L_)_4–6_@Au are determined, which are substantially smaller than those for (Rh_2_L)_*n*_@Au with *n* < 4 (Fig. 4). For the wires with 1,2-bis(4-pyridyl)ethane bridges, the attenuation factor of 0.003 Å^–1^ is close to the smallest *β* values in the hopping regime reported before.^22^,^28^,^64^ Collectively, the wire series with larger π-conjugated bridges, (Rh_2_L_L_)_*n*_, have smaller *β* factors in both tunneling and hopping regimes, while these wires show generally a weak length-dependence of electrical resistance.

**Fig. 5:**
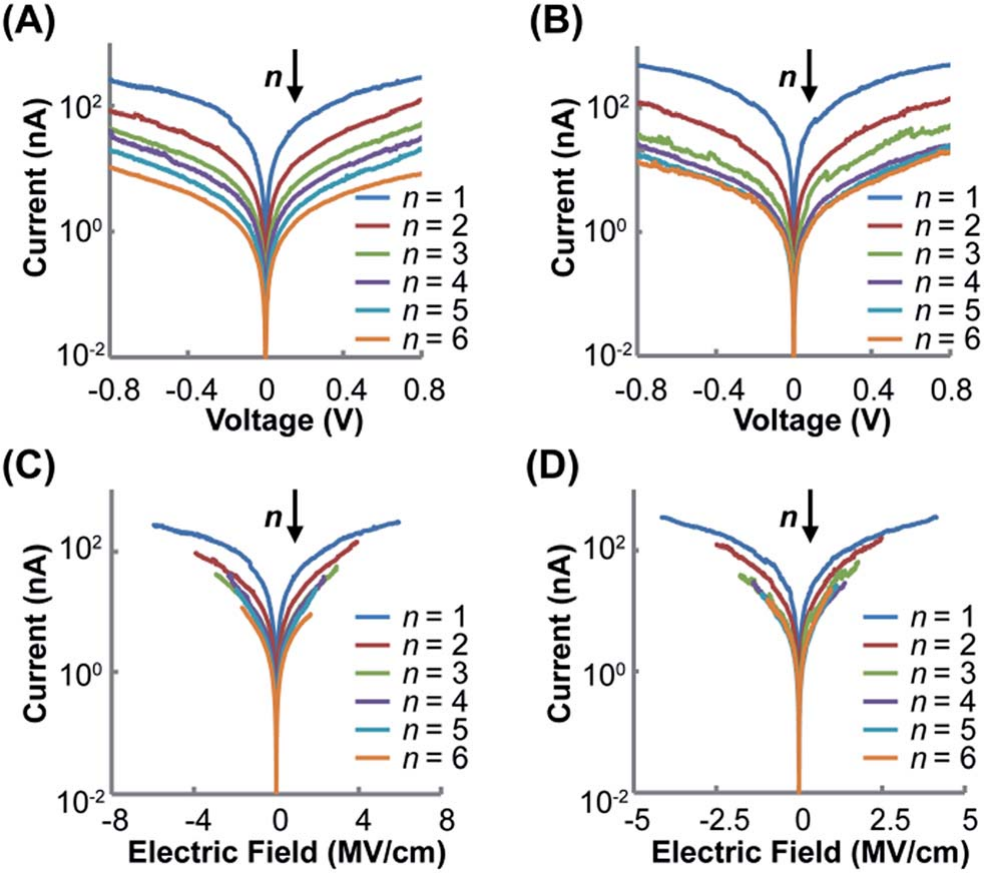
Semi-log plots of the averaged *I*–*V* curves for (Rh_2_L_S_)_*n*_@Au (*n* = 1–6) (A) and (Rh_2_L_L_)_*n*_@Au (*n* = 1–6) (B). Semi-log plots of the averaged *I*–*E* curves for (Rh_2_L_S_)_*n*_@Au (*n* = 1–6) (C) and (Rh_2_L_L_)_*n*_@Au (*n* = 1–6) (D). The deviations from the *I*–*V* and *I*–*E* curves are shown in Fig. S6 (ESI).†

Fig. 5 shows the impacts of voltage (*V*) and electrical field (*E*) on current intensity (*I*) for the two wire series at different lengths. For both (Rh_2_L_S_)_*n*_@Au (Fig. 5A) and (Rh_2_L_L_)_*n*_@Au (Fig. 5B), symmetric *I*–*V* curves are displayed within the testing window (±0.8 V). The *I*–*V* curves for all studied wires with error bars representing the standard deviation are displayed in Fig. S6.† It is found that the current decreases as the wire length increases, consistent with the length dependence of resistance (Fig. 4). Importantly, the two series exhibit the semi-log *I*–*V* curves that can be divided into two groups considering the current variation with respect of the number of the Rh_2_L units (Fig. 5A and B). For short wires (*n* = 1–4), elongating the molecular wires yields a large decrease of the current at all potentials. However, the *I*–*V* curves for the long wires (*n* = 4–6) show smaller current reductions with increasing the wire length. Interestingly, for each of the two wire series, the two groups that show different conductive behaviors are separated by the wire having the same Rh_2_L unit number (*n* = 4), rather than the wire having the same lengths, in accord with the transition point observed in the length dependence of resistance (Fig. 4).

The large voltage-dependence of current for the short wires of both series, as shown by the semi-log *I*–*V* curves (Fig. 5A and B), corresponds to the voltage-driven characteristics for charge tunneling.^17^ On the other hand, in the semi-log *I*–*E* plots, (Rh_2_L_L_)_*n*_@Au (*n* = 4–6) (Fig. 5D) exhibits the traces that collapse nearly on the top of one another. This result reveals that for long wires (Rh_2_L_L_)_*n*_, the charge transport is field driven in nature, corresponding to the hopping mechanism.^17^ The *I*–*E* characteristics observed for (Rh_2_L_L_)_*n*_@Au conform well to the transition from exponential to linear relationships between the resistance (*R*) and the molecular length (*L*), confirming the conversion of the electron transport mechanism from tunneling to hopping. Furthermore, the more homogeneously spaced *I*–*V* (Fig. 5A) and *I*–*E* curves for the pyrazine derived wires (Fig. 5C) are consistent with the mild transition from regime I to II, observed for (Rh_2_L_S_)_*n*_@Au (Fig. 4). These results indicate that while charge transport in (Rh_2_L_S_)_*n*_ is dominated by super-exchange tunneling in regime I, this pathway still plays a significant role in regime II where the hopping mechanism starts to operate. This is understandable from the strong coupling between the Rh_2_ centers and great extent of electronic delocalization for the pyrazine bridged system. The differences in *I*–*V* and *I*–*E* characteristics between (Rh_2_L_S_)_*n*_@Au and (Rh_2_L_L_)_*n*_@Au must originate from their differences in the backbone architecture.

### DFT calculations

As shown above, in regime II (*n* ≥ 4), for wires of the two series having the same number of the bridging ligands, charge hopping behaviors are observed for the wires built by longer bridging ligand L_L_ (1,2-bis(4-pyridyl)ethene), which shows relatively weak Rh_2_–Rh_2_ coupling, as evaluated by the electrochemical analyses. To better understand the experimental results, theoretical calculations at the density functional theory (DFT) level were carried out on the simple models that are not wired to the electrode, aiming at understanding the electronic coupling effects within the molecules. For the regime I, the calculation models are built with two Rh_2_L units (*n* = 2) and a 2-(4-pyridyl) ethanethiol group, that is, (Rh_2_L)_2_(NC_5_H_4_CH_2_CH_2_S) (L = L_S_ or L_L_), as an example of the shorter wires. In the hopping regime, the models of the Rh_2_L moieties, Rh_2_(O_2_CCH_3_)_4_(C_5_H_5_N) (Rh_2_L_S_) and Rh_2_(O_2_CCH_3_)_4_(NC_5_H_4_CHCHC_5_H_4_N) (Rh_2_L_L_), were adopted considering that the hopping sites are relevant to the fragment orbitals but not necessarily to the MOs of the entire molecule.

**Fig. 6:**
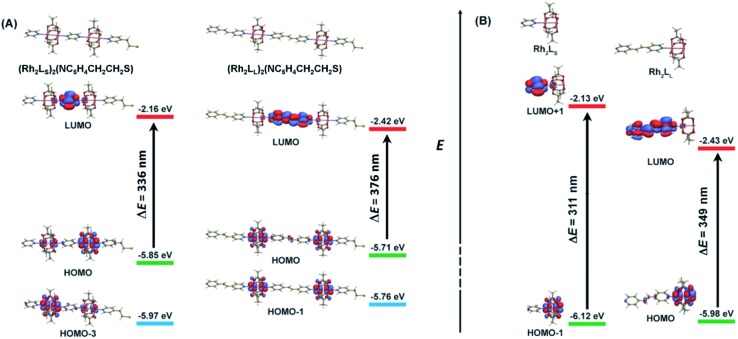
(A) Calculated frontier orbitals and the orbital energies for the Rh_2_ dimers (Rh_2_L)_2_, (Rh_2_L_S_)_2_ (left), and (Rh_2_L_L_)_2_ (right). (B) Calculated molecular orbitals and energies for metal (Rh_2_) to ligand (L) charge transfer for the Rh_2_L moieties in the linear molecules, Rh_2_L_S_ (left), and Rh_2_L_L_ (right).

As shown in Fig. 6, the computational results show that one of the two Rh–Rh π anti-bonding orbitals (
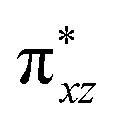
 and 
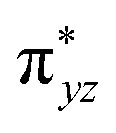
) is symmetrically related to the π orbitals of the conjugated bridging ligand, topologically analogous to the orbital interactions between the ethynyl and phenyl groups in ethynyl benzene. Therefore, for both series, a π orbital interaction is invoked through the π*(Rh_2_)–π*(L) orbital interactions. In both of the Rh_2_ dimer and Rh_2_L fragment cases, the compositions of the MOs predict that the HOMOs of the molecular wires are composed of the π*(Rh_2_) orbitals, while the LUMOs are constructed mainly from the π*(L) orbitals. For the Rh_2_ dimers, calculations also show that the longer bridging ligand gives lower LUMO energy for the wire, *e.g.*, –2.42 eV and–2.16 eV for the Rh_2_ dimers with longer and shorter bridges, respectively. The HOMOs result from “phase out” combination (π* – π*) of the π*(Rh_2_) orbitals for the pyrazine derivative, but “phase in” combination (π* + π*) for the other. The HOMO–LUMO gap for the pyrazine bridged Rh_2_ dimer is 3.69 eV (336 nm), larger than that for the Rh_2_ dimer with the longer bridge by 0.4 eV (40 nm) (Fig. 6), consistent with the experimental observation (45 nm). The calculations on the two models give similar HOMO energies, *i.e.*, –5.85 and –5.71 eV for the molecules with shorter and longer bridges (Fig. 6A), respectively.

It is remarkable that the computational results are in excellent agreement with the ionization energies (IE) for the corresponding Rh_2_ oligomers, *i.e.*, 5.98 and 5.88 eV determined by XPS (Fig. S7†). Therefore, experimental and theoretical results indicate that in both of the two Rh_2_ wire systems, the HOMO is below, but very close to, the gold Fermi level (–5.31 eV).^75^ Given this energetic alignment of the HOMO with the electrode Fermi level, sufficiently strong electronic coupling between the molecule and the electrode is maintained, which facilitates the charge injection from the electrode to the molecule. Since the optically determined HOMO–LUMO gaps can be significantly reduced in the metallic junction,^9^ strong electronic coupling is ensured in the molecular junctions for both of the two wire systems, as evidenced by the large hysteresis of the reduction potential for the (Rh_2_L)_*n*_@Au (*vide supra*).

The calculations also convey important information on the electronic coupling within the molecules. For (Rh_2_L_S_)_2_(NC_5_H_4_CH_2_CH_2_S), the counterpart of the HOMO (π* – π*) is HOMO-3 (π* + π*), as shown in Fig. 6A. These two metal-based MOs are separated in energy by 0.12 eV. Remarkably, in XPS, two peaks at 5.98 eV and 6.18 eV were observed (Fig. S7†), corresponding to the removal of the valence electrons from these metal-based MOs. In contrast, for (Rh_2_L_L_)_2_(NC_5_H_4_CH_2_CH_2_S), the energy difference between the HOMO (π* + π*) and HOMO-1 (π* – π*) is only 0.05 eV and XPS exhibits a single peak at 5.88 eV, consistent with the calculated value (–5.71 eV). These MOs are non-degenerate due to the mediation of the bridging ligand, and the larger the spacing, the stronger the electronic coupling. Thus, the energy difference between the MO counterparts is a measurement of the strength of Rh_2_–Rh_2_ electronic coupling, similar to the equatorially bridged Mo_2_ dimers where the magnitude of the energy gap between (δ + δ) and (δ – δ) has been used to evaluate the metal–metal coupling strength.^43^ Therefore, evidently, the Rh_2_–Rh_2_ coupling in the pyrazine bridged wires is appreciably stronger than that in the 1,2-bis(4-pyridyl)ethene bridged system, consistent with the experimental results. For the latter, the HOMO–(HOMO–1) gap of 0.05 eV indicates that the metal–metal interaction is relatively weak,^43^ being in the charge localized regime.

Calculations on the fragment models show that the HOMO and LUMO are contributed mainly by the Rh_2_ center and bridging ligand orbitals, respectively (Fig. 6B). This π*(Rh_2_)–π*(L) interaction is transformed by the HOMO → LUMO electronic transition in Rh_2_L_L_. For Rh_2_L_S_, such a π*(Rh_2_)–π*(L) interaction is represented by the HOMO–1 → LUMO+1 transition. Therefore, these transitions correspond to the metal to ligand charge transfer (MLCT) absorptions in the electronic spectra (*vide supra*). As shown in Fig. 6B, the π*(Rh_2_) (HOMO) energy for Rh_2_L_L_ is higher than that (HOMO–1) for Rh_2_L_S_, which is in agreement with its lower redox potential (Fig. 3 and Table 1). In addition to the higher π*(Rh_2_) orbital energy for Rh_2_L_L_, the π*(L) orbital energy is substantially low in comparison with that for Rh_2_L_S_. Therefore, the two Rh_2_L fragments differ in the energy gap between these Rh_2_-based and L-based MOs. The metal to ligand charge transfer energy for the Rh_2_L_S_ and Rh_2_L_L_ fragments is calculated to be 3.99 eV (311 nm) and 3.55 eV (349 nm), respectively. The calculated transition energies are in good agreement with the observed MLCT band energies in the UV-vis spectra for the SAMs on the gold substrate, 285 nm for (Rh_2_L_S_)_*n*_@Au and 330 nm for (Rh_2_L_L_)_*n*_@Au, as shown in Fig. 2.

### Interpretation of charge transport behaviors

Given the experimental and theoretical results for the two wire series, the conducting behaviors and the charge transport mechanisms of the systems may be elucidated under the McConnell super-exchange formalism,^58^,^59^ which is widely accepted for the study of the intramolecular electron transfer in solution and proposed in the molecular junction.^76^ In a D–B–A molecular system, according to the super-exchange mechanism, reducing the energy gaps between the donor and bridge and/or the bridge and acceptor benefits directly the charge transfer from the D (A) site to the bridge or *vice versa*, consequently accelerating the D → A electron transfer.^45^,^58^,^59^ Similarly, here, better charge transport characteristics are observed for (Rh_2_L_L_)_*n*_@Au due to the smaller optical gaps. From the lower π* (L_L_) orbital energies for this series, as shown in Fig. 6, higher energy filled π(L_L_) orbitals are anticipated, which would help hole hopping *via* a ligand to metal transition (LMCT). Therefore, under the super-exchange formalism, charge transport between two neighboring Rh_2_ centers in both tunneling and hopping pathways is favored for the (Rh_2_L_L_)_*n*_ wires. On the other hand, in (Rh_2_L_S_)_*n*_, the extra Coulomb repulsion caused by the short Rh_2_–Rh_2_ distance would resist the charge hopping between the adjacent Rh_2_ centers. Therefore, the large optical gap and strong electrostatic effects provide the pyrazine bridged wires with poorer redox conduction in the hopping regime. In the tunneling regime, however, similar charge transport characteristics are observed for (Rh_2_L_S_)_*n*_ and (Rh_2_L_L_)_*n*_ (Table 1). It appears that the increased coupling effects resulting from the shorter charge transfer distance and delocalization for (Rh_2_L_S_)_*n*_ are offset by the larger HOMO–LUMO gap and electrostatic repulsion. Therefore, our results show that the generally defined electronic coupling within the molecule is not an impetus to drive the charge transport under the condition of bias voltage, and accordingly, the extent of electron delocalization may not be used as an effective criterion for the assessment of charge transport characteristic in molecular junctions. Of course, better electronic delocalization in (Rh_2_L_S_)_*n*_@Au should be helpful for efficient charge tunneling; this is why for this series, the attenuation factor *β* in the hopping regime (*n* > 4) is close to that for the shorter wires (*n* < 4), showing the tunneling characteristics. Finally, it should be addressed that when electrons traverse the molecules through bonds in a short distance, both super-exchange and resonance pathways may be involved. For example, as shown in Fig. 6, resonant tunneling may occur between the two non-degenerate metal-based HOMOs. However, it is hard to account for the contributions made by each of them. Therefore, the use of terminology super-exchange tunneling in this context does not preclude the resonant effects.

As is noticed, electron delocalization does not extend throughout the entire long π conjugated wire;^17^ for wires with a sufficiently long length, charges are unevenly distributed along the wires. Therefore, redox exchange, for which the presence of charge localized redox centers is the perquisite, has been considered in diverse molecular junctions.^77^ The study of a single electron transistor reveals that several distinct redox states in the phenylenevinylene oligomer (3.2 nm in length) can be reached with small addition energies, which controls the charge transport properties.^35^ According to Nishihara, different redox states for the [Fe(tpy)_2_] sites in the wire, generated after electron injection from the gold electrode, are responsible for the intra-wire hopping between the [Fe(tpy)_2_] sites along the wire.^28^ The hypothesis of mixed-valence state exchange for redox molecular conduction junctions is also supported by recent theoretical work.^78^ In this study, the experimental observations and computational data lead us to propose that under a bias voltage the Rh_2_ redox sites in the wires are in mixed-valence states, a reduced Rh_2_^II/II^(D) and oxidized Rh_2_^II/III^(A), and redox exchange between two adjacent Rh_2_ centers occurs as follows:

3 –(Rh_2_^II/II^–L–Rh_2_^II/III^)– → –(Rh_2_^II/III^–L–Rh_2_^II/II^)–

For shorter wires (Rh_2_L)_*n*_@Au (*n* < 4) in the tunneling regime, the super-exchange may cross several D–B–A units, being extended to the electrodes by the orbital interactions. For long wires in (Rh_2_L)_*n*_@Au (*n* > 4), the Rh_2_L complex unit serves as the hopping site to transport the charge carriers in the hopping regime.

**Fig. 7:**
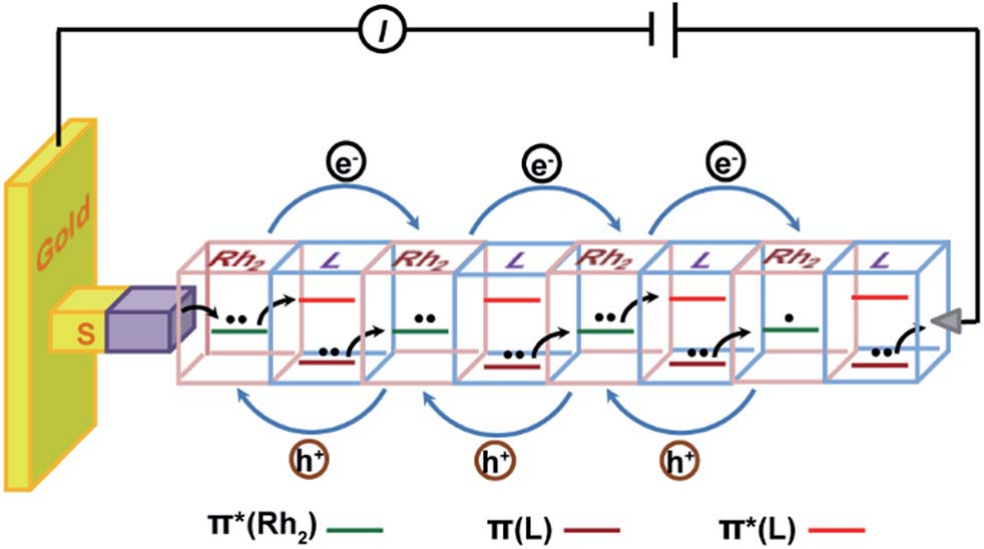
Schematic description of the hopping mechanism for (Rh_2_L)_*n*_ (*n* > 4) wires in the electrode–molecule–electrode junction. Under a bias voltage, the bridged Rh_2_ centers in the wire are in the mixed-valence states. The bridging ligand L provides a low-lying empty orbital (π*) and high-lying filled orbital (π) for electron hopping and hole hopping pathways by the super-exchange mechanism, respectively. Simultaneous and consecutive electron self-exchange between the neighboring redox sites generates redox conductivity in the electrical circuit.

A microscopic description for this hopping mechanism is schematized in Fig. 7, showing charge transport through multiple redox hopping steps under a certain bias. We assume that the mixed-valance states are in the localized regime and there is not any instant charge occupation on the bridge. As shown in Fig. 7, the wire ends with a bridging ligand which is in contact with the conducting probe. Upon application of a bias voltage, migration of the charge carriers starts with the first injection of charge (hole) from the electrodes, creating the first MV Rh_2_^II/II^–L–Rh_2_^II/III^ unit close to the electrode.^9^,^56^ Within a Rh_2_^II/II^–L–Rh_2_^II/III^ unit, Rh_2_ → Rh_2_ electron transfer proceeds through bridge-mediated electron and hole hopping pathways, or electron/hole super-exchange reactions, in which the bridging ligand is involved by providing a low lying empty π* orbital and high lying filled π orbital.^38^ By this hypothesis, the super-exchange tunneling to hopping transition is controlled by the number of Rh_2_–L–Rh_2_ units, instead of the wire length. Experimentally, we observed that for both wire series, the mechanistic transition occurs in the wire with two Rh_2_–L–Rh_2_ units (*n* = 4), but with different lengths. In the situation of molecular junctions, electron self-exchange is not an isolated redox event occurring in a single Rh_2_–L–Rh_2_ unit. Simultaneous and concerted actions of all the Rh_2_–L–Rh_2_ units along the wire generate the redox conductivity in the electrical circuit (Fig. 7). This mechanism conforms well to a previous theoretical study, which proposes a conduction channel dominated by electron localization at the redox centers.^78^

The hopping mechanism proposed here is based on the super-exchange formalism. The metal–ligand interactions, as indicated by the HOMO–LUMO energy difference (optical gap) of the Rh_2_L unit, govern the electron transfer across the bridge. This optical gap can be referred to as the energy barrier for electron hopping between two bridged Rh_2_ centers.^79^ Notably, this is different from organic π conjugated wires, in which the hopping barrier is determined by structural conformation factors.^17^ Our results show that localized systems with small optical gaps present high performance of electrical conductance. These results are parallel with the observations on π-conjugated organic wires constructed with a redox active donor (tetrathiafulvalene, TTF) and acceptor (pyromelliticdiimide, PMDI), which show an enhanced hopping transport with respect to the homogeneous wire systems.^17^,^20^ It is noted that this oligo-tetrathiafulvalene-pyromelliticdiimide-imine (OTPI) system is fully charge localized because of the large internal potential difference (redox asymmetry) between the D and A sites, but has the wire conductivity nearly two orders of magnitude higher than that of the charge delocalized oligophenyleneimine (OPI) wires.^17^,^20^ In both of the two examples of organic and metal–organic redox systems, the hopping efficiency is controlled by the HOMO–LUMO gap, as predicted by the CNS model proposed by Creutz, Newton and Sutin based on the McConnell super-exchange formalism.^80^ However, in the asymmetrical organic D–B–A wires, the HOMO–LUMO gap corresponds to the potential difference between the D and A sites. Differently, for the symmetrical, metal–organic redox wires under investigation, the optical gap is correlated with the differences in orbital energy between the MOs of the redox center and the bridge ligand.

## Conclusion

Through axial coordination, the dirhodium complex [Rh_2_(O_2_CCH_3_)_4_] with *N*,*N*′-bidentate ligands was fabricated linearly and alternately on Au substrates, developing two series of highly ordered π conjugated wires (Rh_2_L_S_)_*n*_ (L_s_ = pyrazine) and (Rh_2_L_L_)_*n*_ (L_L_ = 1,2-bis(4-pyridyl)ethene) (*n* = 1–6). With the Rh_2_ redox centers incorporated into the molecular backbones, these two metal–organic hybrid wire systems exhibit generally weak length dependence of electrical resistance due to the good alignment of the molecular HOMO with the gold Fermi level in energy and the π–π conjugation between the Rh_2_ unit and the bridging ligand. Analyses of the current (*I*)–voltage (*V*) characteristics reveal that in both series, a transition of the charge transport mechanism from super-exchange tunneling to hopping occurs in wires of *n* = 4, disregarding the wire lengths. Surprisingly, smaller attenuation factors (*β*) of electric resistance against increasing the length are found for (Rh_2_L_L_)_*n*_ with longer bridging ligands in both tunneling (*β* = 0.044 Å^–1^) and hopping regimes (*β* = 0.003 Å^–1^), although in (Rh_2_L_S_)_*n*_ the metal–metal interactions are much stronger. This unusual phenomenon is rationalized by two coupling effects that diminish the charge transport ability of the molecules (Rh_2_L_S_)_*n*_ with small bridging ligands, that is, the relatively high MLCT gap and the charge delocalization that imposes strong electrostatic repulsion between the hopping sites. These results suggest that in these wires, the Rh_2_L units function as the hopping sites and the optical gap, corresponding to the HOMO–LUMO energy difference, accounts for the hopping barrier. This hypothesis is supported by DFT calculations on the Rh_2_L motifs, which define the π(Rh_2_)–π(L) orbital interaction and confirm the spectral assignments to the Rh_2_ → L electronic transition. On this basis, it is proposed that under a bias voltage, the redox sites in the wire are in mixed-valence states and simultaneous and consecutive electron (or hole) self-exchange across the bridging ligand generates redox conductivity in the circuit. This work indicates that localized redox active wires with small optical gaps exhibit excellent long-distance conductance. The obtained understanding opens the door for the development of highly conductive molecular wires.

## Materials and methods

### Materials

1,2-bis(4-pyridyl)ethene, pyrazine, rhodium(ii) chloride trihydrate, and tetrabutyl ammonium hexafluorophosphate (^*n*^Bu_4_NPF_6_), 2-(4-pyridyl) ethanethiol, ethanol, ether and dichloride methane were obtained from commercial sources and used without further purification. The tetraacetate dirhodium(ii) were synthesized according to a literature method.^81^

### Preparation of gold films

Three types of gold films were used in this study. A transparent gold film was used for UV-vis spectroscopy study, gold on quartz was used for electrochemical measurements and ultra-flat gold was used for AFM characterization.

### Preparation of transparent gold film and gold film on quartz

First, quartz plates (1 × 4.5 cm^2^, thickness 0.2 cm) were washed using piranha solution (concentrated sulfuric acid: 30% hydrogen peroxide = 2 : 1) followed by water and ethanol sequentially and dried in N_2_. Second, a 3–5 nm thick Ti layer was deposited on the plate by magnetron sputtering. Finally, a 20 nm thick Au layer was deposited on the Ti layer by magnetron sputtering. For electrochemical measurements a 100 nm Au layer was deposited on the Ti layer by magnetron sputtering.

### Preparation of the ultra-flat gold film

Freshly cleaved mica sheets (5 × 5 cm^2^) were placed onto a stainless steel sample holder in a high vacuum evaporator (Model TRP-450 Sky Technology Development, Shenyang, China). Gold (99.999%, Alfa Aesar, Ward Hill, MA) was evaporated at 3 Å s^–1^ until 150–200 nm thickness was reached at a pressure around 7 × 10^–6^ Torr.^82^,^83^ Then, ultra-flat gold with large global flatness is prepared on glass according to the method developed by Hegner *et al.*^84^ and Wagner *et al.*^85^ First, microscope cover slips with a diameter of 12 mm were washed using piranha solution followed by water and ethanol sequentially and dried in N_2_. Second, these cover slips were glued with Epotek 377 (Epoxy Technology, Billerica, MA) onto the gold thin films. After bring the cover slips in contact with the gold thin film, the samples were annealed at 150 °C for 2 hours, allowing the glue to cure. The cover slip was peeled off from the mica substrate with a gold film glued on it prior to use.

### Preparation of metal–organic hybrid SAMs on gold substrates

First, SAMs of 2-(4-pyridyl) ethanethiol were prepared by soaking gold films in 0.01 mM 2-(4-pyridyl) ethanethiol in ethanol for 1 hour at room temperature.^40^ The SAMs were rinsed with ethanol and dried by nitrogen blowing. The fabrication of the metal–organic hybrid oligomers on these SAMs involves two repeating steps. The first step was soaking the SAMs in a 0.2 mM Rh_2_ ethanol solution at –15 °C for 1 hour.^40^ Second, these samples were rinsed with ethanol and dried by nitrogen blowing, followed by soaking in a 0.1 mM solution of the bridging molecules (pyrazine or 1,2-bis(4-pyridyl)ethene) in ether at room temperature for 10 minutes. After taking out from the solutions, the samples were rinsed with ethanol and dried in a nitrogen stream followed by repeating the two steps shown above. After the first cycle, soaking times for both solutions were reduced to 2 minutes.

### Physical measurements

#### UV-vis spectroscopy

UV-vis spectra were measured with a Shimadzu UV-3600 UV-vis-NIR spectrophotometer.

#### Electrochemical measurements

Electrochemical measurements were carried out using a CH instruments model CHI 660D electrochemical analyzer in a 1.0 M ^*n*^Bu_4_NPF_6_ solution in CH_2_Cl_2_ with the studied SAMs on gold as a working electrode, a Pt plate as a counter electrode, and an Ag/AgCl reference electrode.

The surface coverage values of Rh_2_L_L_ and Rh_2_L_S_ units were estimated by the integration of the oxidation peak areas. This integration gives the number of electrons transported from the SAMs' terminal to a gold electrode (*Q*). Therefore, *Γ* can be estimated using the following equation:4
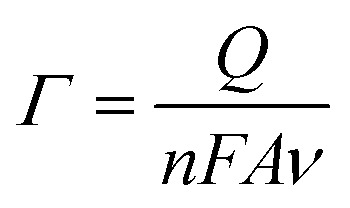
where *n* is the stoichiometric number of electrons involved in the redox reaction, *F* is the Faraday constant, *A* is the electrode area and *ν* is the scan rate.^66^

#### Junction formation and current–voltage measurements by CP-AFM

The *I*–*V* measurements were conducted according to the method reported in the literature.^20^ Molecular junctions were formed by bringing a Pt/Ir coated tip (SCM-PIC-V2 probes, Bruker) into contact with a monolayer. These experiments were performed with a Bruker Innova AFM (Bruker, SO#47233) in a glovebox (Vigor SG1200/750TS). Minimal load force (∼1 nN) to give stable *I*–*V* curves was used to make reproducible contact. We have examined the current–voltage (*I*–*V*) characteristics of the junctions over ±0.8 V. The low voltage resistance was determined from the linear *I*–*V* relationship within the range of ±0.4 V. Three Pt/Ir-coated AFM tips were used for the measurements. The three tips were used separately to examine three sets of junctions: (Rh_2_L_L_)_1–4_@Au (tip 1), (Rh_2_L_L_)_1–6_@Au, (Rh_2_L_S_)_1_@Au (tip 2), and (Rh_2_L_L_)_2_@Au, (Rh_2_L_S_)_2–6_@Au (tip 3). In order to reduce the systematic errors introduced by changing tips, each measurement of junctions (Rh_2_L)_1–4_@Au was conducted using at least two different tips to confirm that similar resistances were obtained. For each junction, at least 30 *I*–*V* curves over more than five sample points were collected.

#### AFM imaging and AFM based nanoshaving

AFM topographic imaging and AFM based nanoshaving were conducted using an Innova AFM (Bruker, SO#47233) in a glovebox (Vigor, SG1200/750TS). AFM imaging and nanoshaving were performed with a silicon nitride cantilever with a spring constant of 0.01 N m^–1^ (MSNL-10) and 0.35 N m^–1^ (RTESP-300), respectively.

#### XPS spectra

The XPS spectra of (Rh_2_L_S_)_*n*_ and (Rh_2_L_L_)_*n*_ oligomers were recorded on a Thermo Scientific ESCALAB 250Xi spectrometer with an Al Kα X-ray (1486.8 eV) source using a hemispherical analyzer in an ultrahigh vacuum (<2 × 10^–9^ mbar) system and the X-ray anode was operated at 150 W. The binding energy is referenced to the work function of 4.41 eV for the instrument.

### Wire length calculations

The wire lengths were estimated from the X-ray single crystal structures of the Rh_2_L units that construct the wires. An Rh_2_L_L_ unit is 13.6 Å ^47^ in length and an Rh_2_L_S_ unit is 7.4 Å.^48^ The length of the molecule 2-(4-pyridyl) ethanethiol on the gold surface is 6.9 Å.^49^

### DFT calculations

All calculations were run using Gaussian 09 programs (revision A.01). For all calculation models, the geometry was optimized for the neutral states at a DFT level using the B3LYP functional and CPCM (conductor-like polarizable continuum model) solvent model (dichloromethane) in conjunction with the LANL2DZ basic set for rhodium and 6-31G for other atoms. Post-processing for visualization of the molecular orbitals generated by the DFT calculations was performed using VMD and POV-Ray programs. The calculated results are summarized in Fig. S8.†

## Conflicts of interest

There are no conflicts to declare.

## Supplementary Material

Supplementary information
