# Supplementary material for: Understanding the charge transport properties of redox active metal–organic conjugated wires
Source: Chem Sci. 2018 Feb 19;9(14):3438–50. doi: 10.1039/c7sc04727d (PMC5934749; doi:10.1039/c7sc04727d)
Supplement: Supplementary file 1 [file SC-009-C7SC04727D-s001.pdf]

Electronic Supplementary Material (ESI) for Chemical Science.
This journal is © The Royal Society of Chemistry 2018

# Supporting Information for

## Understanding the Charge Transport Properties of Redox Active Metal-Organic Conjugated Wires

Donglei Bu, Yingqi Xiong, Ying Ning Tan, Miao Meng, Paul J. Low, Dai-Bin Kuang, and Chun Y. Liu*

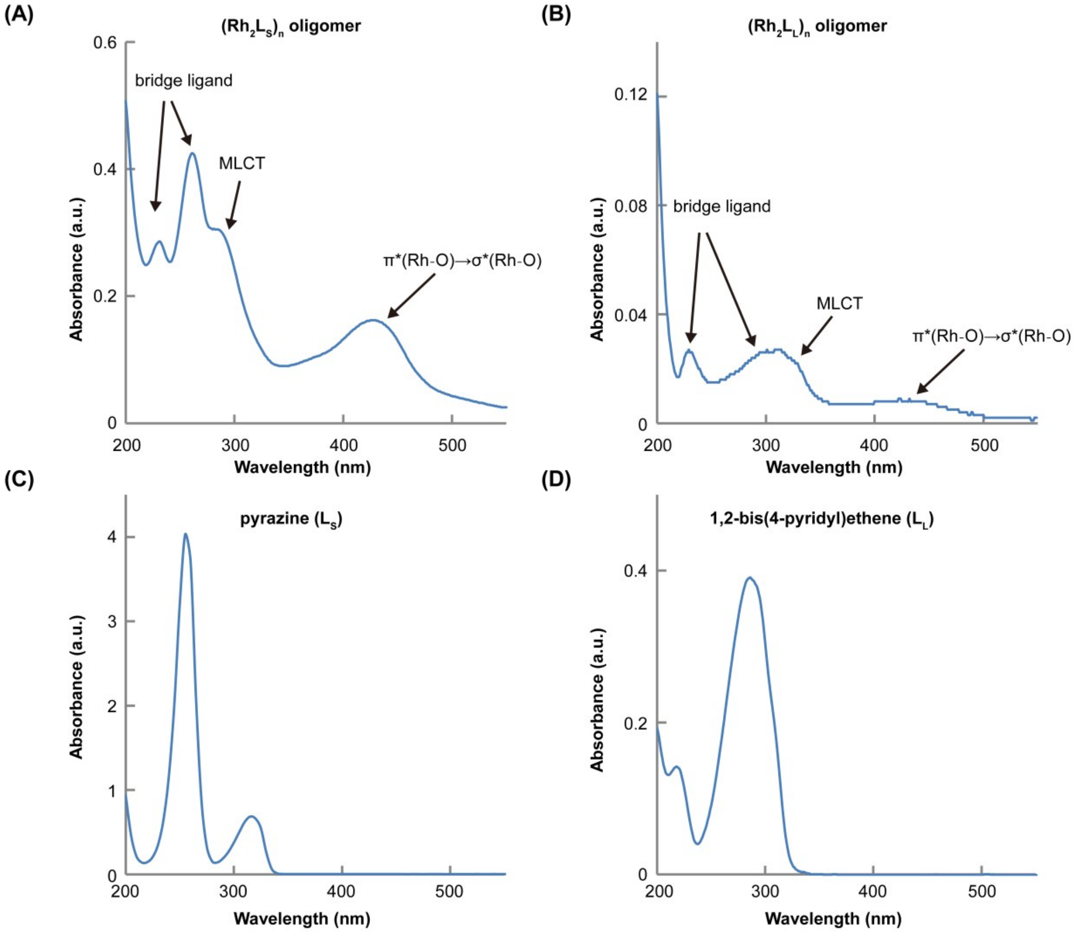


**Figure S1.** UV-Vis spectra of oligomers $(Rh_2L_S)_n$ (A) and $(Rh_2L_L)_n$ in ethanol (B), and free ligands pyrazine (C) and 1,2-bis(4-pyridyl)ethene ($L_L$) (D) in dichloromethane (1.0 mM). The $(Rh_2L_S)_n$ and $(Rh_2L_L)_n$ oligomers were prepared by mixing equal moles of $Rh_2(OCCH_3)_4$ with the corresponding bridging ligand pyrazine ($L_S$) or 1,2-bis(4-pyridyl)ethene ($L_L$) in ethanol. Orange solid product formed immediately, which was isolated by filtration and dried in vacuum. The ethanol saturate solutions of $(Rh_2L_S)_n$ and $(Rh_2L_L)_n$ were used for the spectroscopic measurements.

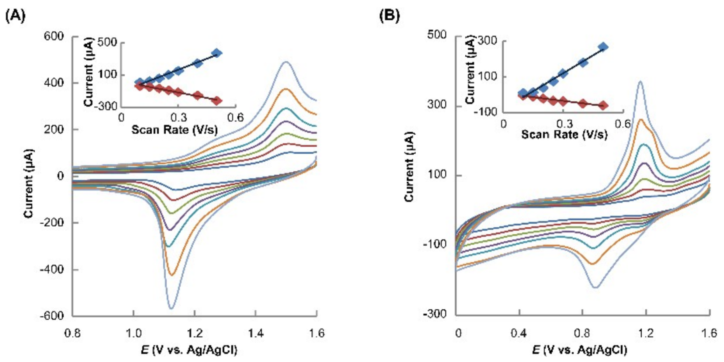


**Figure S2.** Cyclic voltammograms of $(Rh_2L_S)_6$@Au (A) and $(Rh_2L_L)_6$@Au (B) with scan rates of (from inside to outside) 0.10, 0.15, 0.20, 0.25, 0.30, 0.40, 0.50 V/s. Insert plots show the variation of $i_{pa}$ and $i_{pc}$ *vs.* scan rate, indicating that the peak current increases linearly with scan rate. These results confirm that the wires are surface bonded.

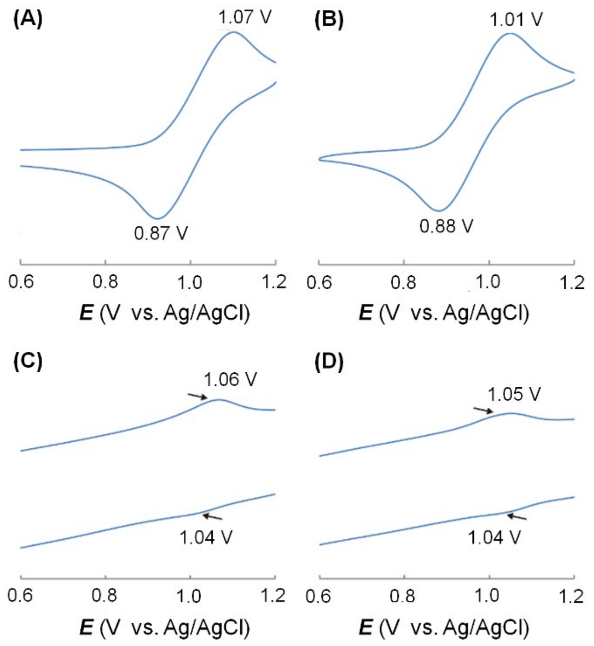


**Figure S3.** Cyclic voltammograms of the reference compounds $Rh_2(O_2CCH_3)_4(NC_5H_5)_2$ (A) and $Rh_2(O_2CCH_3)_4(NC_5H_5C_2H_2C_6H_6)_2$ (B) in dichloromethane. Compounds $Rh_2(O_2CCH_3)_4(NC_5H_5)_2$ and $Rh_2(O_2CCH_3)_4(NC_5H_5C_2H_2C_6H_6)_2$ were prepared by mixing $Rh_2(OCCH_3)_4$ with two eqv. of pyrazine ($NC_5H_5$) or 1,2-bis(4-pyridyl)ethene ($NC_5H_5C_2H_2C_6H_6$) in dichloromethane. After 5 min stirring, the reaction solution turned to red. To this solution, $^{n}Bu_4NPF_6$ (final concentration of 1.0 M) was added and then, the CVs were recorded. The half wave redox potential for $Rh_2^{II/II} \rightarrow Rh_2^{II/III}$ process is determined to be 0.97 V for $Rh_2(O_2CCH_3)_4(NC_5H_5)_2$ and 0.95 V for $Rh_2(O_2CCH_3)_4(NC_5H_5C_2H_2C_6H_6)_2$. Cyclic voltammograms (CVs) for $(Rh_2L_S)$@Au (n = 1) (C) and $(Rh_2L_L)$@Au (n = 1) (D). For both, the half wave redox potential for $Rh_2^{II/II} \rightarrow Rh_2^{II/III}$ process is determined to be 1.05 V with an anodic and cathodic splitting of 10 mV, which is agreement with the potentials for the $Rh_2$ monomers in solution.

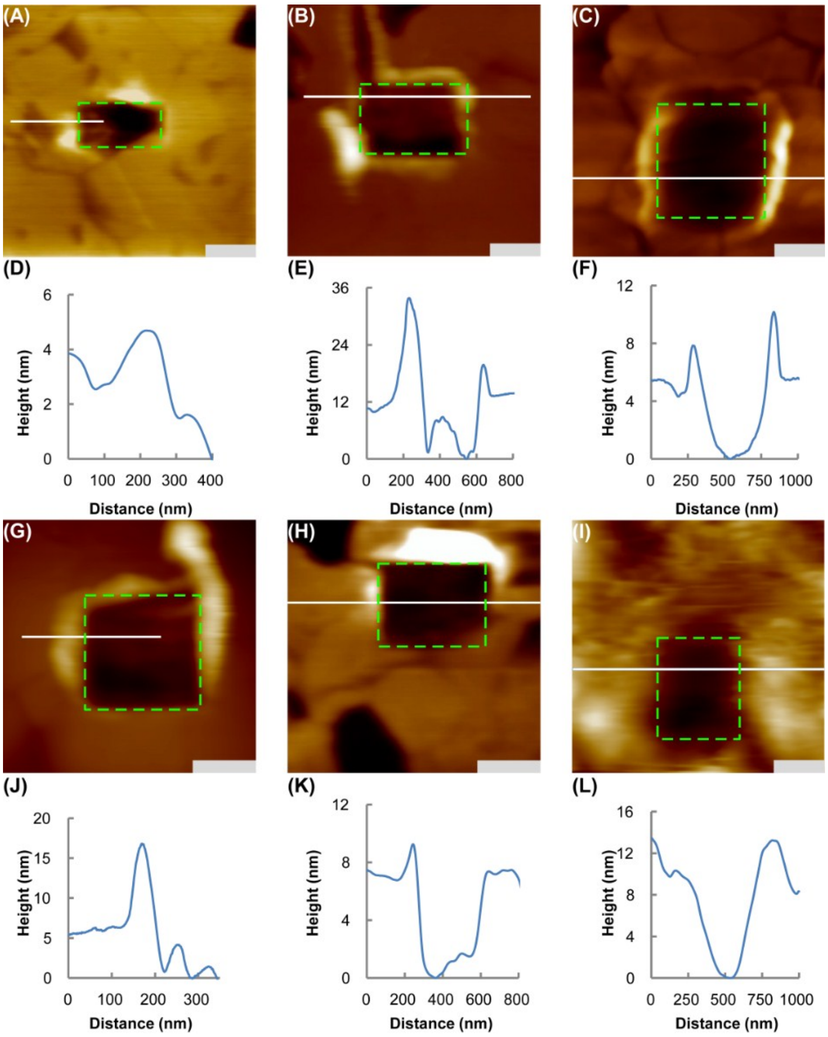


**Figure S4** AFM topographic images of monolayers of fabricated wires and their corresponding nanoshaved regions (green squares) for $(Rh_2L_S)_2$@Au(A) and $(Rh_2L_S)_4$@Au (B), $(Rh_2L_S)_6$@Au(C), $(Rh_2L_L)_2$@Au(G), $(Rh_2L_L)_4$@Au(H) and (I) $(Rh_2L_L)_6$@Au. For wires $(Rh_2L_S)_n$@Au ($n$ = 2, 4 and 6), the corresponding cursor profiles are presented in figure (D), (E) and (F), respectively, and for$(Rh_2L_L)_n$@Au ($n$ = 2, 4 and 6), the corresponding cursor profiles are shown in figure (J), (K) and (L), respectively. Scale bars = 200 nm. By removing the implanted molecules on the gold surface, the difference in height between the shaved region and surrounding areas were measured, which is taken as the thickness (T) of the SAMs (see the text). For $(Rh_2L_S)_n$@Au, with increasing $n$ from 2 to 6, the height increases from 2.04 ± 0.29 to 4.74 ± 0.18 nm, whereas the height of the $(Rh_2L_L)_n$@Au SAMs rises from 3.08 ± 0.64 nm ($n$ = 2) to 8.78 ± 0.53 nm ($n$ = 6).

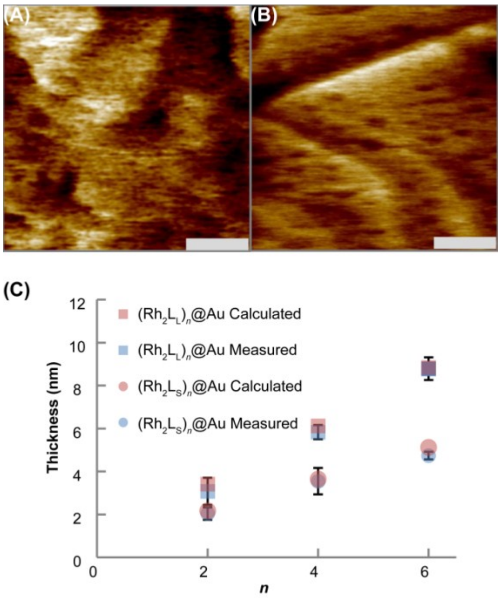


**Figure S5.** AFM topographic images of $(Rh_2L_S)_6$@Au (A) and $(Rh_2L_L)_6$@Au (B). Scale bars = 20 nm. The large bright areas indicate morphologically the assembled structural domains, in which homogeneous layer structures are formed. (C) Comparison between the measured SAMs thickness (blue) and the calculated wire length (red) for $(Rh_2L_S)_6$@Au (circle) and $(Rh_2L_L)_6$@Au (square). For both series, the measured SAMs' heights are in good agreement with the wire lengths (L) estimated from the X-ray structures of similar structural motifs. The consistency indicates that the SAMs were implanted on the Au surfaces in a "standing-up" manner.

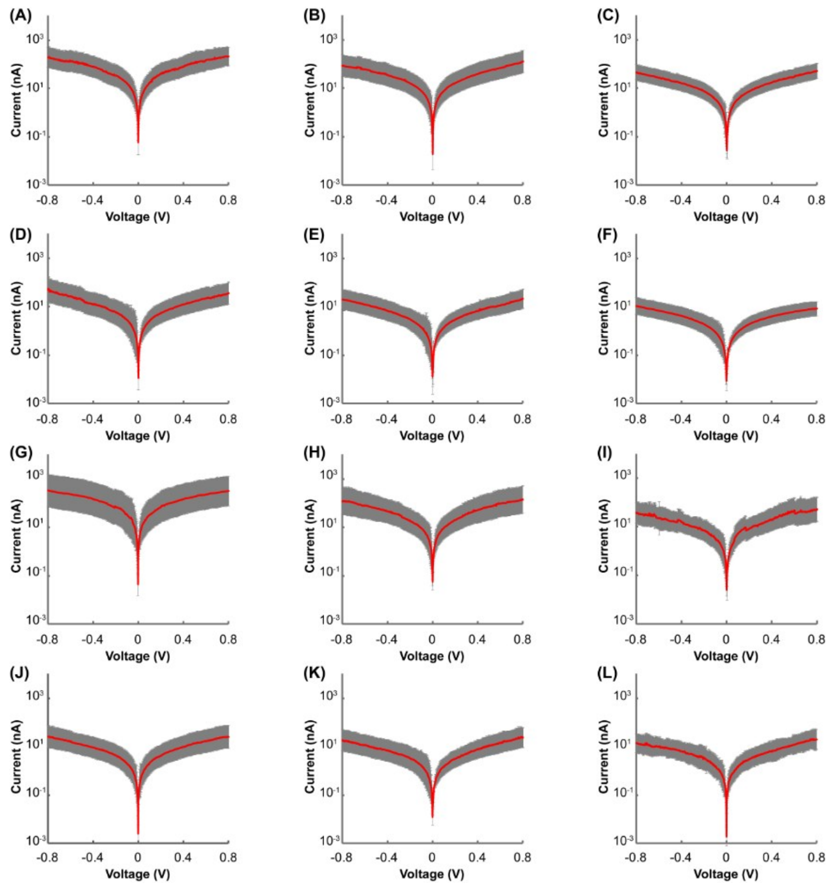


Figure S6. Semilog plots of the average $I$–$V$ curves built from at least 30 individual curves for with error bars $(Rh_2L_S)_n$@Au (n =1-6) (A - F), and $(Rh_2L_L)_n$@Au (n =1-6) (G - L). The grey error bars represent the standard deviation.

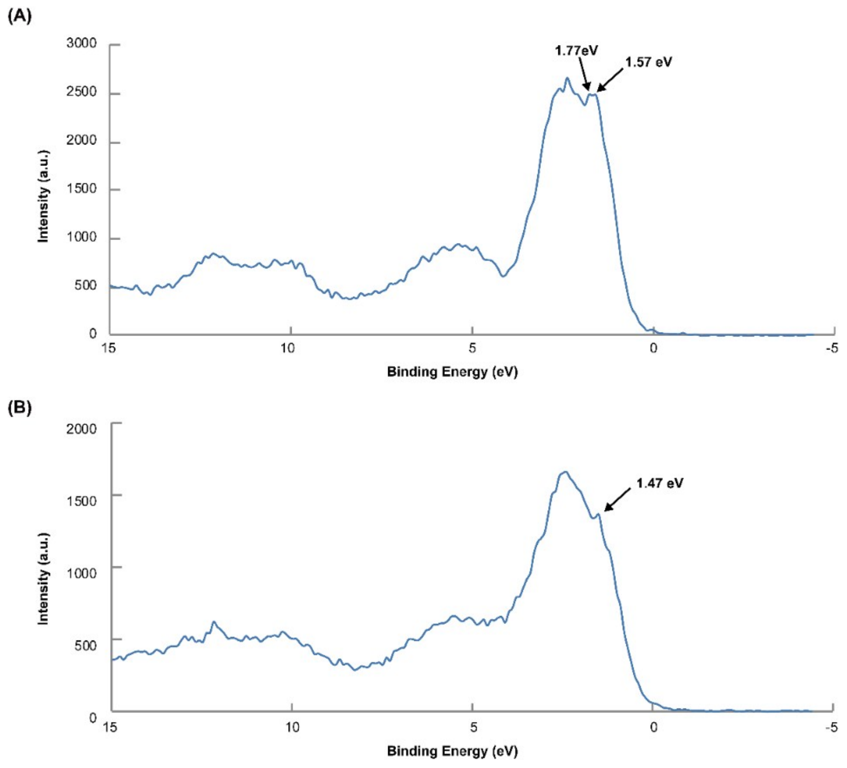


**Figure S7.** XPS spectra of the oligomers $(Rh_2L_S)_n$ (A), and $(Rh_2L_L)_n$ (B). The samples were prepared as described above (Figure S1, caption). The binding energy is referred to the work function of 4.41 eV for the instrument (Thermo SCIENTIFIC ESCALAB 250Xi). The ionization energies of the molecule were estimated by sum of the binding energy of the concerned peak with the Fermi energy (4.41 eV). For $(Rh_2L_S)_n$, the low energy peaks at 1.57 eV and 1.77 eV are attributed to removal of the valence electrons from the $Rh_2^{4+}$ centers in the oligomer, corresponding to the δ-based HOMO and HOMO-3 (see the text); accordingly, the ionization energies are determined to be 5.98 eV and 6.18 eV. For $(Rh_2L_L)_n$, the ionization energy of the $Rh_2^{4+}$ centers is 5.88 eV determined from the peak (1.47 eV), in excellent agreement with the δ-based HOMO and HOMO-1 energies (see the text).

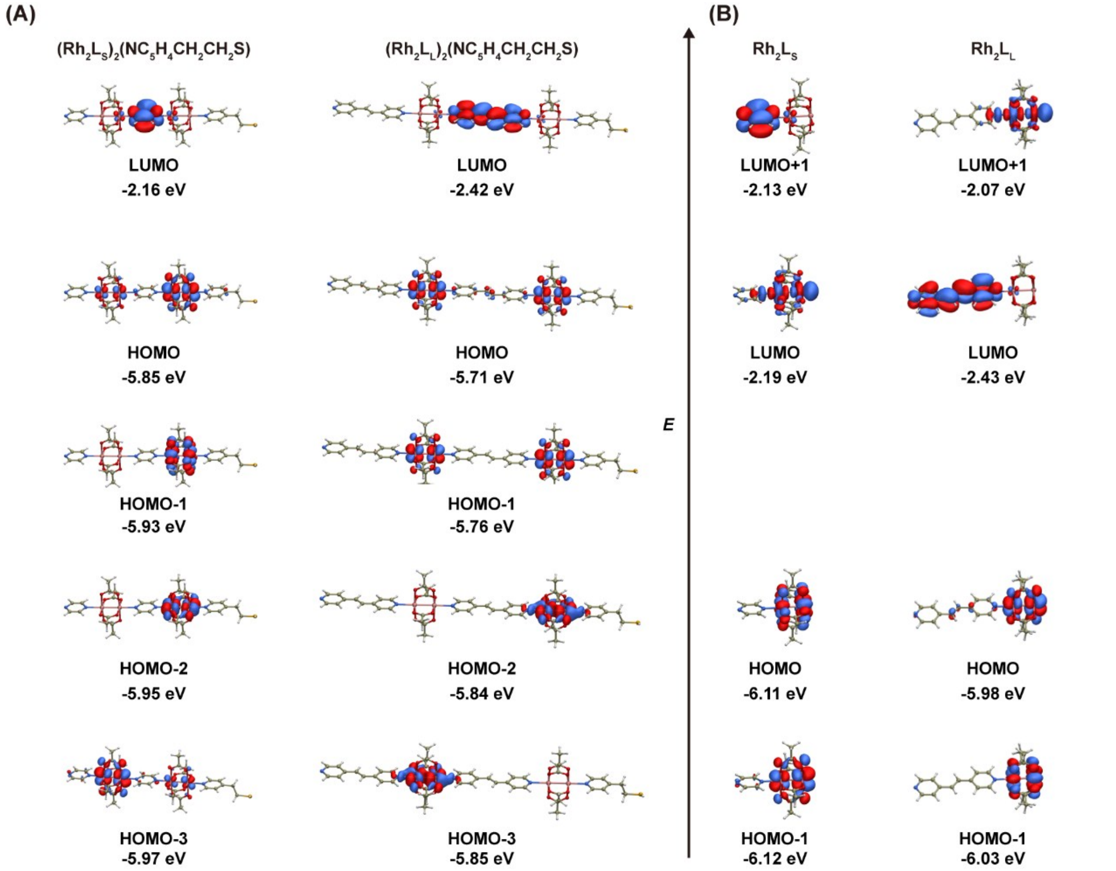


**Figure S8**. (A) Calculated frontier molecular orbitals and the orbital energies for the $Rh_2$ dimers, $(Rh_2L_S)_2$ (left), and $(Rh_2L_L)_2$ (right). (B) Calculated frontier molecular orbitals and energies associated with metal to ligand charge transfer for $Rh_2L$ fragments, $Rh_2L_S$ (left), and $Rh_2L_L$ (right).
